# Amino Acid Differences in the 1753-to-1851 Region of TcdB Influence Variations in TcdB1 and TcdB2 Cell Entry

**DOI:** 10.1128/mSphere.00268-17

**Published:** 2017-08-02

**Authors:** Jonathan J. Hunt, Jason L. Larabee, Jimmy D. Ballard

**Affiliations:** Department of Microbiology and Immunology, University of Oklahoma Health Sciences Center, Oklahoma City, Oklahoma, USA; University of Maryland Medical Center

**Keywords:** B2′, *Clostridium difficile*, TcdB1, TcdB2, entry, *tcdB*, toxin B

## Abstract

*Clostridium difficile* TcdB2 enters cells with a higher efficiency than TcdB1 and exhibits an overall higher level of toxicity. However, the TcdB2-specific sequences that account for more efficient cell entry have not been reported. In this study, we examined the contribution of carboxy-terminal sequence differences to TcdB activity by comparing the binding, uptake, and endosomal localization of TcdB1 and TcdB2 or selected recombinant fragments of these proteins. Our findings suggest that sequence differences in the amino acid 1753 to 1851 region proximal to the combined repetitive oligopeptide domain (CROP) support enhanced uptake of TcdB2 and localization of toxin in acidified endosomes. In the absence of this region, the CROP domains of both forms of the toxin exhibited similar levels of cell interaction, while the addition of amino acids 1753 to 1851 greatly increased toxin binding by only TcdB2. Moreover, the amino acid 1753 to 2366 fragment of TcdB2, but not TcdB1, accumulated to detectable levels in acidified endosomes. Unexpectedly, we discovered an unusual relationship between endocytosis and the efficiency of cell binding for TcdB1 and TcdB2 wherein inhibition of endocytosis by a chemical inhibitor or incubation at a low temperature resulted in a dramatic reduction in cell binding. These findings provide information on sequence variations that may contribute to differences in TcdB1 and TcdB2 toxicity and reveal a heretofore unknown connection between endocytosis and cell binding for this toxin.

**IMPORTANCE** TcdB is a major virulence factor produced by *Clostridium difficile*, a leading cause of antibiotic-associated diarrhea. Hypervirulent strains of *C. difficile* encode a variant of TcdB (TcdB2) that is more toxic than toxin derived from historical strains (TcdB1). Though TcdB1 and TcdB2 exhibit 92% overall identity, a 99-amino-acid region previously associated with cell entry and spanning amino acids 1753 to 1851 has only 77% sequence identity. Results from the present study indicate that the substantial sequence variation in this region could contribute to the differences in cell entry between TcdB1 and TcdB2 and possibly explain TcdB2’s heightened toxicity. Finally, during the course of these studies, an unusual aspect of TcdB cell entry was discovered wherein cell binding appeared to depend on endocytosis. These findings provide insight into TcdB’s variant forms and their mechanisms of cell entry.

## Abstract (precis)

TcdB is a major virulence factor produced by *Clostridium difficile*, a leading cause of antibiotic-associated diarrhea. Hypervirulent strains of *C. difficile* encode a variant of TcdB (TcdB2) that is more toxic than toxin derived from historical strains (TcdB1). Though TcdB1 and TcdB2 exhibit 92% overall identity, a 99-amino-acid region previously associated with cell entry and spanning amino acids 1753 to 1851 has only 77% sequence identity. Results from the present study indicate that the substantial sequence variation in this region could contribute to the differences in cell entry between TcdB1 and TcdB2 and possibly explain TcdB2’s heightened toxicity. Finally, during the course of these studies, an unusual aspect of TcdB cell entry was discovered wherein cell binding appeared to depend on endocytosis. These findings provide insight into TcdB’s variant forms and their mechanisms of cell entry.

## INTRODUCTION

*Clostridium difficile* TcdB isoforms TcdB1 and TcdB2 share 92% sequence identity but exhibit distinct differences in antigenicity and cytotoxicity (1). TcdB2 is produced by NAP1/027/BI strains of *C. difficile*, which have been associated with several outbreaks of *C. difficile* infection over the past 15 years (2–4). Compared to TcdB1, TcdB2 is more toxic and appears to cloak epitopes to avoid recognition by neutralizing antibodies (5). Importantly, antibody-mediated neutralization of TcdB reduces disease severity (6), supporting the idea that TcdB is a major virulence factor. Thus, a better mechanistic understanding of how TcdB2 differs from TcdB1 may provide insights into the reasons for the heightened virulence of *C. difficile* NAP1/027/BI strains.

As a single-polypeptide intracellular bacterial toxin, TcdB appears to use an A-B mechanism for cell entry, wherein the B domain supports and mediates the entry of an enzymatic domain into the cell. The enzymatic glucosyltransferase domain (GTD; amino acids 1 to 544) (7) shows 99% identity between TcdB1 and TcdB2, and residues critical for glucosylation do not vary between the two forms of the toxin (1). Unlike the nearly identical GTD, the B region (amino acids 545 to 2366) exhibits 91% identity between TcdB1 and TcdB2, suggesting that differences in antigenicity and cytotoxicity could be due to activities associated with this region of the toxin. Within the B fragment, the cysteine protease autoprocessing domains (amino acids 544 to 767) of TcdB1 and TcdB2 exhibit 97% identity and the pore-forming regions (amino acids 970 to 1107) of the two forms of the toxin are 99% identical. Unlike the autoprocessing and pore-forming domains, the receptor binding and cell entry regions (amino acids 1651 to 2366) of TcdB1 and TcdB2 are 88% identical. Considering the 1651-to-2366 region’s role in cell entry, we have hypothesized that the substantial sequence differences in this portion of the toxin contribute to the variation between the cell entry rates of TcdB1 and TcdB2.

TcdB’s interaction with target cells is mediated by two or possibly three domains through which dual receptor interactions promote intoxication (8–10). Residues 1851 to 2366 include a series of imprecise repeat sequences, commonly referred to as the combined repetitive oligopeptide (CROP) region, that are predicted to bind cell surface glycans (11). Depending on the cell type, truncated forms of TcdB that lack the CROP region retain partial or full activity in cytotoxicity assays, indicating that this domain may be dispensable for cellular intoxication in some situations (12–14). More recently, studies have shown that a region proximal to the CROP domain of TcdA and TcdB is also involved in cell binding and may explain why CROP-deficient forms of the toxin retain cytotoxicity (14–16). Supporting this notion, Yuan and colleagues showed that the region spanning amino acids 1500 to 1851 of TcdB interacts with chondroitin sulfate proteoglycan 4 (CSPG4) on HeLa cells (9), and LaFrance and colleagues have shown that TcdB can directly bind to poliovirus receptor-like 3 (PVRL3) on Caco-2 cells independently of the CROP region (8). Tao and colleagues recently described the discovery of frizzled (FZD) proteins as receptors for TcdB (10). They also dissected the distinctions between CSPG4 and FZD binding by TcdB. As part of this work, and in contrast to the report of Yuan and colleagues, they found that dual receptor binding occurs when the CROP domain interacts with CSPG4 and a domain proximal to the CROP region interacts with FZDs. Interestingly, FZD binding can be mediated by two fragments of TcdB, amino acids 1 to 1830 and 1501 to 2366. Thus, FZD binding appears to occur within amino acids 1501 to 1830 of TcdB. These studies indicate that TcdB may intoxicate cells through a dual receptor interaction involving distinct interactions mediated by the CROP and non-CROP binding domains of the toxin. Expanding on these analyses, Manse and Baldwin proposed at least three distinct TcdB receptor binding domains utilized to various extents on different cell types and suggested that a third region spanning amino acids 1372 to 1493 also contributes to TcdB cell interactions (17). Collectively, these results suggest that TcdB encodes a complex set of cell binding regions. The extent to which sequence variation between TcdB1 and TcdB2 impacts their ability to engage multiple receptors has not been determined.

In regard to TcdB1 and TcdB2, these findings are of particular interest because we previously reported that sequence variations in the region spanning amino acids 1753 to 1851 (1753–1851 region) of these two forms of TcdB impacted the exposure of neutralizing epitopes and the extent of multimerization of carboxy-terminal fragments of the toxin (5). Whether the conformational effects of the 1753–1851 region also impact cell interactions either directly by influencing receptor binding or indirectly by impacting the conformation of receptor binding regions is not known.

In the present study, we explored the differences in TcdB1 and TcdB2 cell binding and cell entry and the contributions of the region proximal to the CROP domain to these interactions. The findings indicate that TcdB2 is more efficiently taken up from the cell surface and that the 1753–1852 region is essential for this to occur. In the absence of the 1753–1852 region amino acid sequence, the CROP domain of both forms of the toxin had a substantially reduced ability to accumulate in acidified vesicles. Finally, we describe an unusual correlation between cell binding and uptake wherein conditions that disrupt endocytosis dramatically reduce the efficiency of toxin binding to cells.

## RESULTS

### Differential cell association between TcdB1 and TcdB2.

**FIG 1:**
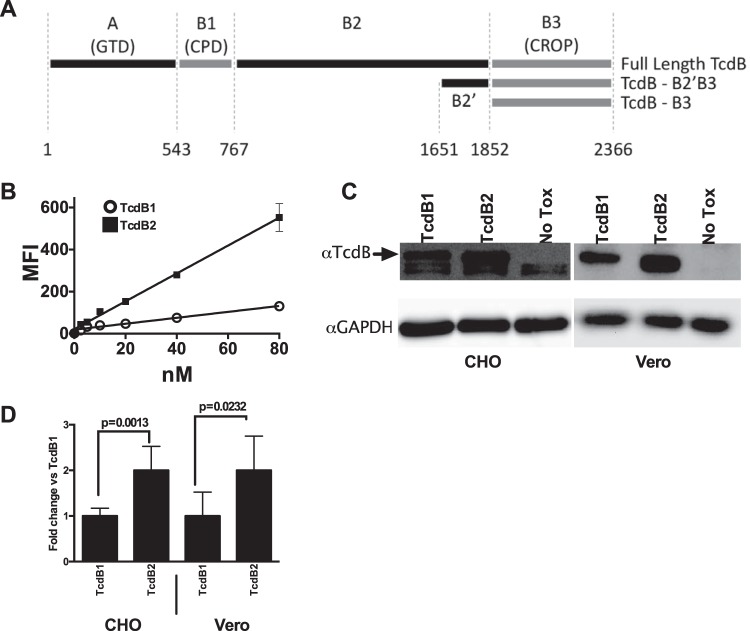
Interaction of full-length TcdB with cells. (A) Cartoon illustrating the TcdB and TcdB fragments used in this study. GTD, glucosyltransferase domain; CPD, cysteine protease domain; CROP, combined repetitive oligopeptides. (B) Summary of flow cytometry of CHO-K1 cells stained with Alexa Fluor 488-labeled full-length TcdB1 (open circles) or TcdB2 (closed squares). *P* < 0.01 for all concentrations of TcdB2 versus TcdB1. MFI, mean fluorescence intensity. (C) Western blotting of whole-cell lysates representative of six independent experiments evaluating the binding of 0.37 nM full-length TcdB1 and TcdB2 to CHO-K1 and Vero cells. GAPDH, glyceraldehyde-3-phosphate dehydrogenase. (D) Quantification by densitometry of the blots in panel C.

The nomenclature for the various TcdB1 and TcdB2 recombinant fragments used in this study is summarized in Fig. 1A. Flow cytometry was used to examine CHO-K1 cell binding of Alexa Fluor 488-derivatized versions of TcdB1 and TcdB2 across concentrations ranging from 2.5 to 80 nM. Results from this experiment performed at 37°C indicated that neither toxin achieved saturable binding and TcdB2 appears to interact with CHO-K1 cells to a greater extent than TcdB1 does (Fig. 1B). As further confirmation of differences between TcdB1 and TcdB2 cell interactions, the next set of experiments used a Western blotting approach to examine binding to CHO-K1 and Vero cells (Fig. 1C). The quantified data are presented in Fig. 1D and confirm what was observed by flow cytometry, that significantly more TcdB2 was able to associate with cells when incubations were carried out at 37°C.

### The B2′B3 region confers differential toxin binding.

**FIG 2:**
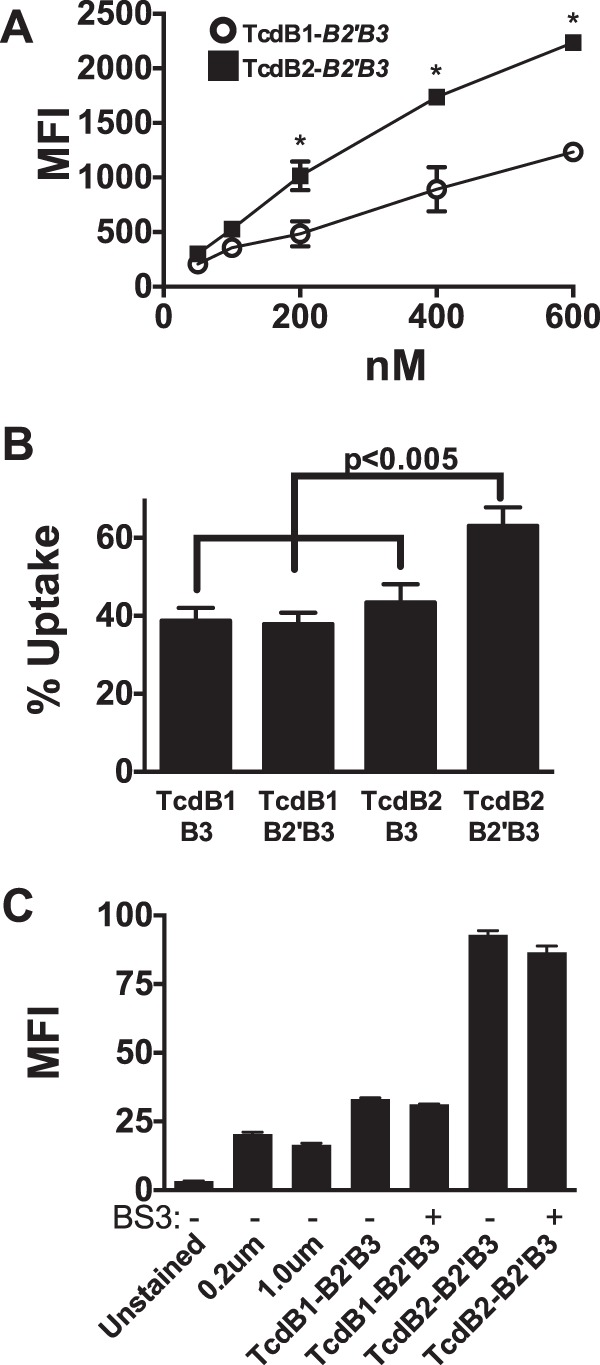
The B2′B3 region confers the binding phenotype of full-length TcdB. (A) Summary of flow cytometry presented as mean fluorescence intensity (MFI) of CHO-K1 cells stained with Alexa Fluor 647-labeled TcdB1 B2′B3 (open circles) or TcdB2 B2′B3 (closed squares) for 1 h at 37°C. *, *P* < 0.05. (B) Uptake of TcdB truncations by CHO-K1 cells exposed to a 400 nM concentration of Alexa Fluor 488-labeled TcdB-B2′B3 or TcdB-B3 fragments for 1 h at 37°C and then analyzed by flow cytometry. The fluorescence that was not quenched by trypan blue represents the amount of B2′B3 that was taken up by the cells. The bar graph compares the percentages of B2′B3 and B3 taken up by CHO-K1 cells. (C) Summary of binding controls carried out with and without chemical cross-linking (BS^3^) of Alexa Fluor 488-labeled B2′B3 fragments used to stain CHO-K1 cells (200 nM). Two different fluorescein isothiocyanate-labeled microspheres were also included as controls for the uptake of large complexes.

We then asked if the B2′B3 region of TcdB could confer the differential binding observed with full-length TcdB1 and TcdB2. Recombinant B2′B3 from TcdB1 and TcdB2 was labeled with Alexa Fluor 647, and interaction with CHO-K1 cells was evaluated by flow cytometry at protein concentrations ranging from 50 to 600 nM. By this analysis, the B2′B3 fragment from TcdB2 was found to exhibit significantly higher levels of cell binding than the corresponding TcdB1 fragment when incubations were performed at 37°C (Fig. 2A).

Trypan blue has been used to discriminate between extracellular and intracellular populations of large particles and bacterial toxins because of its ability to quench extracellular membrane-associated fluorescence (18–20). Using trypan blue as the quencher in the next set of experiments, we determined the relative intracellular and extracellular levels of fragments derived from TcdB1 and TcdB2. As shown in Fig. 2B, approximately 40% of TcdB1-derived B3 and B2′B3 fragments were internalized (Fig. 2B). A similar level of TcdB2 B3 was internalized. In contrast, almost 60% of TcdB2-derived B2′B3 was internalized by CHO-K1 cells, suggesting that the B2′ region of TcdB2, but not that of TcdB1, enhances cell entry.

### Oligomerization of B2′B3 alone is not sufficient to confer increased binding.

We have previously reported that the B2′B3 region of TcdB2 can form oligomers while the same region of TcdB1 does not (5). This raised the possibility that TcdB2 B2′B3 interacts with cells more robustly because of the size of the complex. To address this, cross-linked large complexes of TcdB1 B2′B3 were generated by the previously reported method (5) and their cell interaction was compared with that of TcdB2 B2′B3. We also examined binding by 0.2- and 1.0-μm fluorescent microspheres, again to determine if the results of the assay were simply a reflection of cell interactions by particles of different sizes. Though bis(sulfosuccinimidyl)suberate (BS^3^) cross-linking generated multimeric complexes of TcdB1 B2′B3 (data not shown), they did not interact with cells more efficiently than the uncross-linked form of this fragment (Fig. 2C). Nor did the large particles interact with cells with a higher efficiency than either of the TcdB fragments. Thus, the greater binding of TcdB2 B2′B3 than TcdB1 B2′B3 does not appear to be related to the formation of higher-order complexes. Taken together, these data suggest that TcdB2 is more efficient at associating with cells and that the B2′B3 region alone confers increased binding, as well as internalization.

### The B3 region does not compete for binding with B2′B3.

**FIG 3:**
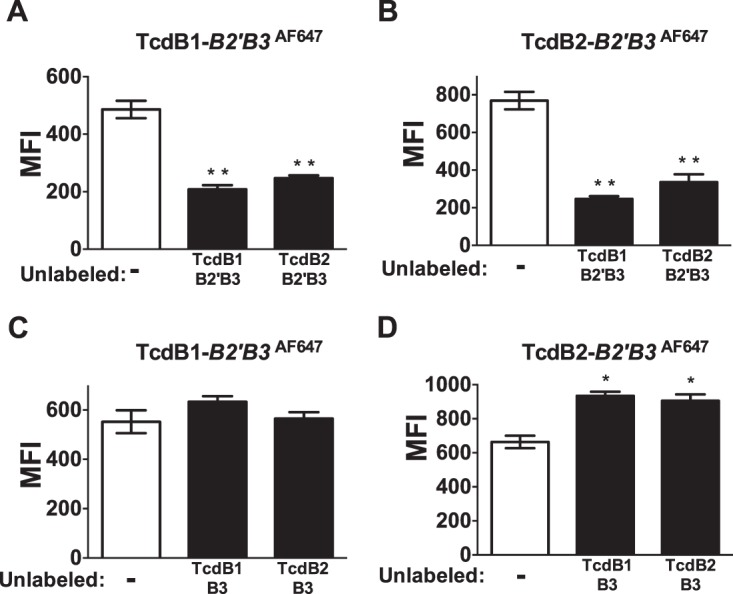
Binding competition between B2′B3 and B3 fragments of TcdB. Bar graphs represent quantified flow cytometry data shown as mean fluorescence intensity (MFI). CHO-K1 cells were exposed to 100 nM Alexa Fluor 647-labeled TcdB1 B2′B3 or Alexa Fluor 647-labeled TcdB2 B2′B3 for 1 h at 37°C. (A, B) Exposures performed in the presence or absence of a 10-fold molar excess of unlabeled TcdB1 B2′B3 or TcdB2 B2′B3. (C, D) Exposures carried out with and without a 10-fold molar excess of unlabeled TcdB1 B3 or TcdB2 B3. *, *P* < 0.01; **, *P* < 0.001.

B2′B3 and B3 fragments derived from both forms of TcdB were used in reciprocal competition experiments to examine their relative specificity. When CHO-K1 cells were incubated with Alexa Fluor dye-labeled TcdB1 B2′B3, binding was reduced by including a 10-fold molar excess of unlabeled B2′B3 from both TcdB1 and TcdB2 (Fig. 3A). The reverse was also true, as B2′B3 from TcdB1 and TcdB2 competed for binding of Alexa Fluor 647-labeled TcdB2 B2′B3, indicating that this region of the toxin could reciprocally compete for cell binding (Fig. 3B). However, unlike the B2′B3 region, the B3 region alone did not compete for binding (Fig. 3C and D). These data suggest that the B3 region may be dispensable for competitive inhibition or adopts an unfavorable structure in the absence of the B2′ sequence.

### Effective interaction of TcdB with cells correlates with endocytosis.

**FIG 4:**
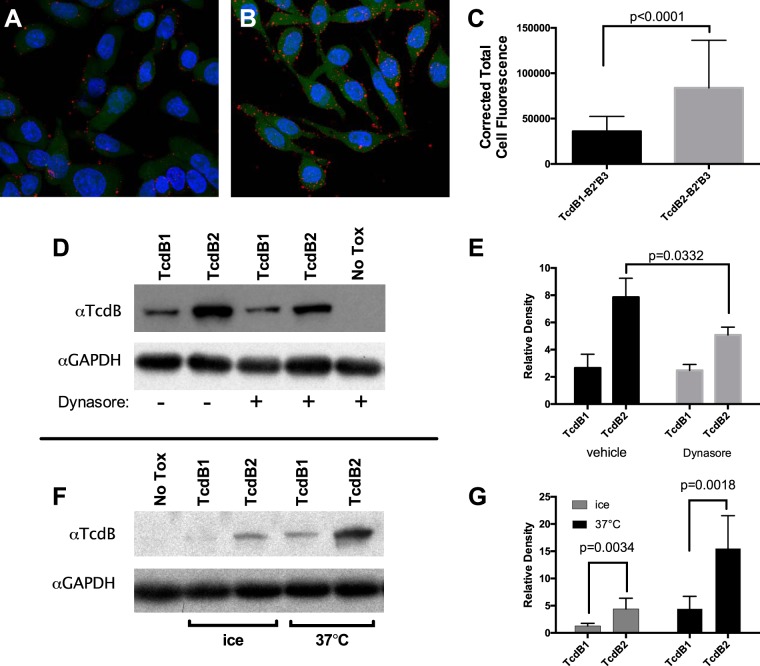
Effective TcdB cell interactions correlate with endocytosis. (A, B) Representative confocal maximum-intensity projections of CHO-K1 cells stained with 250 nM Alexa Fluor 647-labeled TcdB1 B2′B3 (red) (A) or Alexa Fluor 647-labeled TcdB2 B2′B3 (red) (B). Ten micromolar calcein AM (green) was used to counterstain the cytoplasm of live cells with intact membranes, and 0.5 μg/ml Hoechst 33258 (blue) was used to visualize the nucleus. (C) Quantification of the images in panels A and B, expressed as corrected total cell fluorescence. (D) Representative immunoblotting of full-length TcdB1 and TcdB2 associated with CHO-K1 cells in the presence or absence the dynamin inhibitor dynasore (80 μM). Incubations were carried out for 30 min at 37°C. (E) Quantification of data presented in panel D, expressed as relative band density. (F) Representative immunoblotting of full-length TcdB1 and TcdB2 associated with CHO-K1 cells when incubations were carried out for 30 min at a temperature that permits (37°C) or inhibits (ice) endocytosis. (G) Quantification of data presented in panel F, expressed as relative band density. GAPDH, glyceraldehyde-3-phosphate dehydrogenase.

Confocal microscopy was next used to visualize the cellular localization of B2′B3 derived from TcdB1 and TcdB2. Consistent with the data presented in Fig. 2, approximately 2.3-fold less Alexa Fluor 647-labeled TcdB1 B2′B3 signal associated with CHO-K1 cells (Fig. 4A), compared to the signal that was observed from Alexa Fluor 647-labeled TcdB2 B2′B3 (Fig. 4B). Quantification of the corrected total cell fluorescence from multiple frames is presented in Fig. 4C. Because TcdB2 and TcdB2 B2′B3 exhibit a more robust interaction with cells and more TcdB2 B2′B3 than TcdB1 B2′B3 was internalized, the potential role of endocytosis in the overall cell association was determined. TcdB1 and TcdB2 were incubated with CHO-K1 cells that had been pretreated with the dynamin inhibitor dynasore or the control vehicle (dimethyl sulfoxide [DMSO]), and the total cellular interaction was measured by immunoblot analysis of lysates prepared from these treated cells. In the presence of the endocytosis inhibitor dynasore, TcdB2 binding to cells was significantly reduced (Fig. 4D and E). Because endocytosis is also inhibited by a low temperature (21), we incubated TcdB1 and TcdB2 with CHO-K1 cells either on ice or at 37°C. The results are presented in Fig. 4F and G and closely mirror what we observed by inhibiting endocytosis with dynasore. Additionally, when incubations were carried out on ice or with dynasore, more TcdB2 than TcdB1 was bound to cells, indicating that the increased association of TcdB2 could be a result of more efficient internalization, as well as more efficient binding.

### TcdB2 B2′B3 localizes to acidified compartments.

**FIG 5:**
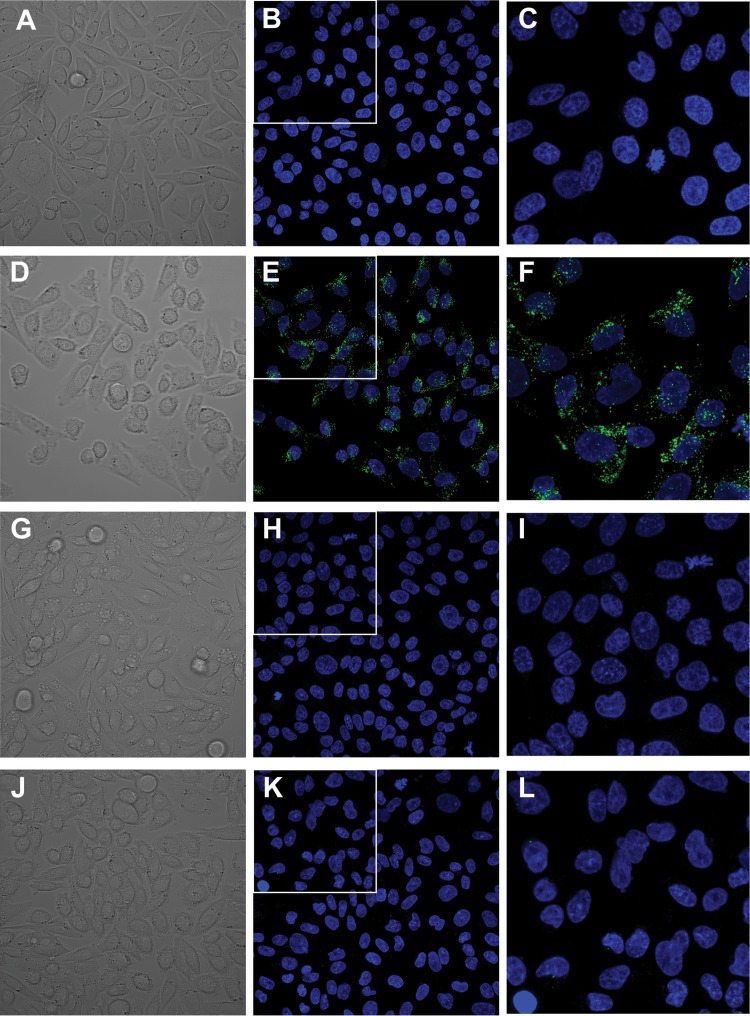
TcdB2 B2′B3 localizes to acidified compartments. Transmitted fluorescence and maximum-intensity projections of pHrodo-labeled TcdB fragments (green) (used at 250 nM) incubated with CHO-K1 cells for 1 h at 37°C. DNA was stained with 0.5 µg/ml Hoechst 33258 (blue). (A to C) TcdB1 B2′B3 incubated with the vehicle control. (D to F) TcdB2 B2′B3 incubated with the vehicle control. (G to I) TcdB2 B2′B3 incubated with 80 μM dynasore. (J to L) TcdB2 B3 incubated with the vehicle control. Panels A, D, G, and J show transmitted fluorescence. Panels C, F, I, and L are ×2 magnifications of the inserts in panels B, E, H, and K, respectively.

Next, the localization of TcdB fragments within cells was investigated. These fragments were labeled with the acid pH-sensitive dye pHrodo and incubated with CHO-K1 cells. Live-cell images were captured by confocal microscopy at 37°C. We found that 100% of the cells treated with the pHrodo-labeled TcdB2 B2′B3 fragment exhibited robust fluorescence (Fig. 5D to F), while no cells incubated with pHrodo-labeled TcdB1 B2′B3 had a detectable pHrodo signal (Fig. 5A to C). pHrodo-labeled TcdB2 B2′B3 was also incubated with cells in the presence of dynasore to further confirm the effect of this inhibitor. As expected, the pHrodo signal was dramatically reduced in the presence of dynasore (Fig. 5G to I). Finally, the contribution of TcdB2 B2′ was confirmed by examining the localization of TcdB-B3 alone in acidified compartments. As shown in Fig. 5J to L, in the absence of B2′, the remaining TcdB2-derived pHrodo-labeled B3 region is clearly deficient in the ability to accumulate in acidified vesicles. This suggests that B2′ from TcdB2 may be more effective at promoting uptake and entry of the toxin into an acidified compartment.

## DISCUSSION

TcdB1 and TcdB2 are two variants of the same toxin that exhibit differences in antigenicity and toxicity (1, 22). As part of a continuing effort to pinpoint the critical differences between these two forms of the toxin, we examined the role of carboxy-terminal sequence variations on cell association and cell entry. Working to the advantage of this study is the fact that sequence variation is not extensive (92% identity) and some regions, such as the B2′ region (77% identity), are clearly more different than other regions of TcdB1 and TcdB2. The B2′B3 region of TcdB1 did not exhibit a similar level of cell entry (Fig. 2B) nor did it appear to accumulate in endocytic vesicles in a manner like that observed for the same portion of the toxin found in TcdB2 (Fig. 5).

The 1753–1851 region continues to be of interest and growing importance in understanding functional regions of TcdB. Our previous work found that this region contributed to multimer formation (5) and that converting amino acids in this region of TcdB1 to the corresponding amino acids found in TcdB2 increased multimerization efficiency in the B2′B3 region of TcdB1. In line with the importance of this region, Zhang et al. found that a 97-amino-acid deletion in an area that closely overlaps B2′ prevents translocation (15). Considering that the B3 fragment of TcdB2 does not accumulate in acidified endosomes, it is reasonable to conclude that the 1753–1852 region supports more efficient penetration of cells by this form of TcdB.

To date, three receptors for TcdB have been described. The first, CSPG4, appears to be essential for TcdB intoxication of the different cell types at a lower toxin concentration. CSPG4 appears to bind TcdB in a region just encompassing the 1753–1851 domain and could be a critical target of the B2′ portion of TcdB1 and TcdB2 (9). It is very possible that B2′ variants differ in their interactions with CSPG4 and that this directly affects interactions of TcdB1 and TcdB2 with target cells. A second receptor, PVRL3, has also been found to mediate TcdB interactions with target cells (8). Unlike CSPG4, PVRL3 appears be expressed on intestinal epithelial cells and might also serve as a receptor mediating differential interaction between TcdB1 and TcdB2 (8, 23). A third receptor family, FZD, is also expressed in the colonic epithelium and binds to TcdB independently of the B3 region (10). Thus, like interactions with CSPG4, interactions with FZD proteins could also increase the efficiency of TcdB2 cell entry.

Our results indicate that treating cells with dynasore not only prevents TcdB endocytosis but also dramatically reduces the overall interaction of TcdB with the cell. As an inhibitor of dynamin, dynasore prevents the constriction and closure of endocytic vesicles generated in clathrin-coated pits (24). Dynasore and other inhibitors of clathrin-dependent endocytosis have been shown to block TcdB cellular intoxication (25). However, dynasore is also known to impact other aspects of membrane biology, including the depletion of cholesterol and alteration of membrane microdomains (26), and it is possible that disrupting these events contributes to reduced binding by TcdB. However, the notion that cell binding and endocytosis might not be two distinct events in TcdB intoxication is further supported by our data showing a large difference in cell binding at 37°C and on ice, the latter of which prevents endocytosis (21). It is possible that a low temperature reduces receptor levels at the cell surface by altering exocytosis, but in either case, this is an unusual characteristic among bacterial toxins. For example, *Bacillus anthracis* protective antigen binds to cells at 4°C in a way sufficient to form channels and support pH-induced translocation (27). Evaluation of cell binding by cholesterol-dependent cytolysins is also routinely measured by flow cytometry at 4°C (28, 29). Notably, TcdA itself has been shown to bind to hamster brush border membranes more effectively at 4°C than at 37°C, and this is the basis of carbohydrate binding by the toxin (30). Though more work is needed in this area, these data suggest that TcdB may not reside on the cell surface with a high-affinity interaction strong enough to maintain contact unless the protein is rapidly trapped in endocytic vesicles.

## MATERIALS AND METHODS

### Production of native toxin.

Native TcdB was produced by culturing *C. difficile* (VPI 10463 and BI17 6493) by the dialysis method as previously described (31, 32). From these cultures, supernatants were isolated and TcdA was removed by a thyroglobulin affinity chromatography protocol (32). After TcdA was removed, TcdB was purified by anion-exchange chromatography (Q-Sepharose HP; GE Healthcare) in 20 mM Tris-HCl (pH 8.0) and 20 mM CaCl_2_. This method yields native TcdB with a purity of >95%, as demonstrated by the single 270-kDa band obtained by SDS-PAGE analysis with Coomassie staining.

### Production of recombinant B2′B3 and B3.

Constructs for expression of the B2′B3- and B3-encoding regions of the *tcdB* gene (Fig. 1A) in *Escherichia coli* were generated previously (5). *E. coli* BL21(DE3) (New England Biolabs) was grown to an optical density at 600 nm of 0.8 and induced with 300 µM isopropyl-β-d-thiogalactopyranoside (IPTG) for 16 h at 16°C, and protein fragments were purified by Ni^2+^ affinity chromatography, resulting in proteins >95% pure as determined by SDS-PAGE with Coomassie staining.

### Toxin labeling.

After purification, TcdB1, TcdB2, and carboxy-terminal fragments were labeled on primary amines with various fluorescent dyes (Alexa Fluor 488 or 647 or pHrodo green) to analyze cell interaction via flow cytometry and fluorescence microscopy. To conjugate these dyes with TcdB1, TcdB2, and carboxy-terminal fragments, we used the manufacturer’s protocol for the conjugation reaction, unincorporated dye removal, and quantification of incorporated dye (Molecular Probes). For full-length TcdB, the Alexa Fluor 488 dye incorporation was approximately 6 mol of dye/mol of protein. For TcdB-B2′B3 and TcdB-B3, the Alexa Fluor 488 and 647 dye incorporation was approximately 3 mol of dye/mol of protein. For pHrodo constructs, equivalent amounts of protein were adjusted to pH 4.0 with citric acid sodium phosphate buffer and the amount of fluorescence measured was not different between the constructs.

### Cross-linking.

Cross-linking of TcdB fragments was carried out essentially as described in reference 5. Briefly 35 µM purified protein was incubated with 700 µM BS^3^ (Pierce) at room temperature for 30 min in phosphate-buffered saline (PBS). Excess BS^3^ was inactivated by adding Tris buffer (pH 8) to a final concentration of 40 mM. Cross-linking was confirmed by SDS-PAGE, and then cross-linked and control fragments were used at a final concentration of 200 nM. Control microspheres (sulfate FluoSpheres) were acquired from Thermo (Fisher) Scientific.

### Fluorescence microscopy.

CHO-K1 cells (ATCC) were cultured in F12-K medium with 10% fetal bovine serum (FBS; ATCC) in tissue culture-treated T-75 flasks at 37°C in the presence of 6% CO_2_. These cells were then seeded into eight-well chambered coverglass slides (Nunc) at a density of 2 × 10^4^/well in phenol red-free F12-K containing 10% FBS. After the cells were allowed to attach overnight, they were treated with toxin or toxin fragments. Images were captured on a Zeiss LSM-710 confocal microscope and analyzed with ImageJ to calculate corrected total cell fluorescence (33).

### Flow cytometry.

CHO-K1 cells were cultured in F12-K medium (Life Technologies) with 10% FBS in tissue culture-treated T-75 flasks at 37°C in the presence of 6% CO_2_. To measure interactions between cells and the labeled toxin, cells were detached with a nonenzymatic cell dissociation buffer (Invitrogen) and counted with a hemocytometer. Next, 250,000 cells were suspended in 250 μl of F12-K medium with 10% FBS and exposed to various fluorescently labeled toxins. The cells were incubated at 37°C for 1 h, and then fluorescence was quantified with a FACSCalibur flow cytometer (BD Biosciences). The resulting data were analyzed with FlowJo software (Tree Star). For trypan blue quenching experiments, samples were analyzed as described above with or without the addition of trypan blue to a final concentration of 0.4%.

### Western blotting.

Cells were seeded at 250,000 per well of a 12-well plate. After overnight incubation, plates were further incubated for 15 min at 37°C. Next, the culture medium was replaced with toxin-containing medium and the cells were incubated at the temperatures indicated for 1 h. Cells were washed three times with PBS and harvested in lysis buffer, and 20 μg of total protein was separated by SDS-PAGE and transferred to a polyvinylidene difluoride membrane. The membrane was probed with a 0.1-μg/ml concentration of anti-TcdB antibody AF6246 (R&D Systems) and a horseradish peroxidase (HRP)-conjugated anti-sheep secondary antibody or with a 1:100,000 dilution of anti-glyceraldehyde-3-phosphate dehydrogenase antibody 6C5 (Abcam, Inc.) and an HRP-conjugated anti-mouse secondary antibody. Blots were developed by chemiluminescence with film. The film was imaged with an Alpha Innotech FluorChem Q system equipped with a white light transilluminator.

### Inhibition of endocytosis.

Endocytosis was blocked by pretreatment of cells with 80 µM dynasore (Sigma) for 30 min at 37°C, and DMSO was used as the vehicle control (24). Dynasore was included for the duration of toxin incubation before samples were prepared for immunoblotting or microscopy. Endocytosis was blocked by preincubation of cells on ice for 30 min before toxin addition. Incubations were carried out on ice, and all buffers and reagents were ice cold in preparation for immunoblotting or flow cytometry.

### Statistical analysis.

When appropriate, data from at least three independent experiments were compared by using an unpaired two-tailed *t* test with GraphPad Prism 6.
